# Imaging and clinical features of primary hepatic sarcomatous carcinoma

**DOI:** 10.1186/s40644-018-0171-7

**Published:** 2018-10-12

**Authors:** Dongli Shi, Liang Ma, Dawei Zhao, Jing Chang, Chen Shao, Shi Qi, Feng Chen, Yunfang Li, Xing Wang, Yanyan Zhang, Jing Zhao, Hongjun Li

**Affiliations:** 1 Department of Diagnostic Radiology, Beijing You’an Hospital, Capital Medical University, No.8, Xi Tou Tiao, Youanmen wai, Fengtai District, Beijing, 100069 China; 2 Center of Interventional Oncology and Liver Diseases, Beijing You’an Hospital, Capital Medical University, No.8, Xi Tou Tiao, Youanmen wai, Fengtai District, Beijing, 100069 China; 3 Department of pathology, Beijing You’an Hospital, Capital Medical University, No.8, Xi Tou Tiao, Youanmen wai, Fengtai District, Beijing, 100069 China

**Keywords:** Liver, Sarcomatous carcinoma, Combined hepatocellular-cholangiocarcinoma, Cholangiocarcinoma, Hepatocellular carcinoma

## Abstract

**Background:**

Primary hepatic sarcomatous carcinoma (PHSC) is a rare malignancy composed of both carcinomatous (either hepatocellular or cholangiocellular) and sarcomatous components. The purpose of our study was to evaluate the imaging and clinical findings of PHSCs, improving the understanding and diagnosis of tumors.

**Methods:**

We retrospectively reviewed the imaging and clinical findings of ten patients with pathologically proven PHSCs, including two cases of sarcomatous intrahepatic cholangiocarcinoma (S-ICC), seven cases of sarcomatous hepatocellular carcinoma (S-HCC) and one case of sarcomatous combined hepatocellular and cholangiocarcinoma (S-HCC–CC). Six patients underwent computed tomography (CT) scans and five underwent magnetic resonance imaging (MRI) scans with one of them having both CT and MRI scans.

**Results:**

Eight of ten patients had a background of chronic hepatitis or cirrhosis. The elevation of alpha-fetoprotein (AFP) was positive in half of the patients. All the tumors were located near the liver subcapsular area and six of ten cases were massive with round or oval shapes and ill-defined. The lesion textures were mainly heterogeneous in eight tumors for the necrosis or hemorrhage. Eight tumors showed hypo-enhancement and nine tumors exhibited initial peripheral rim (five cases) or heterogeneous (four cases) enhancement, followed by progressive (six cases) and peripheral or partial washout (three cases) on the later phases. Of the seven surgically resected tumors, five showed liver capsular invasion with one of them rupturing into the perihepatic space. Vascular thrombosis (five cases), intrahepatic metastasis (four cases), adjacent organ invasion or seeding (three cases), and lymph node metastasis (four cases) were found on imaging or in pathology. The follow-up period ranged from one to 36 months. Four patients with T3-T4 staging died from recurrence and metastasis between 2 and 5 months, and three patients with T1 staging did not have any recurrence between 16 and 24 months.

**Conclusion:**

PHSC generally presents as a subcapsular mass with hypovascularity and may be characterized by rim-like or heterogeneous enhancement on the arterial phase and a progressive dynamic pattern. These tumors usually coincide with chronic hepatitis or cirrhosis and poor prognosis appears to be associated with TNM staging.

## Background

Primary hepatic sarcomatous carcinoma (PHSC) is a rare malignancy composed of both carcinomatous (either hepatocellular or cholangiocellular) and sarcomatous components [1]. This entity is differentiated from hepatic carcinosarcoma (CS), which contains both hepatocellular carcinoma and a true heterogonous sarcoma component such as chondrosarcoma, malignant fibrous histiocytoma, osteosarcoma, leiomyosarcoma, fibrosarcoma, rhabdomyosarcoma, and other mesenchymal tumors arising in the liver [1–4]. Various terms have been used to describe these biphasic tumors, including CS, sarcomatoid carcinoma, pseudosarcoma, and spindle cell carcinoma. Sarcomatous carcinoma (SC) and CS, however, are most commonly used and easily confused. Currently, pathologists are still faced with the dilemma of how to distinguish CS from SC when making pathological diagnoses. It is suggested that such mixed tumors should be diagnosed as SC when the sarcomatous component is predominantly composed of spindle cells, but the epithelial cells are still morphologically, immunohistochemically, and ultrastructurally identifiable [1, 5–8].

PHSC is extremely rare, accounting for only 0.2% of the primary malignant liver tumors [1]. The prevalence of sarcomatous hepatocellular carcinoma (S-HCC) and sarcomatous intrahepatic cholangiocarcinoma (S-ICC) is 1.8–9.4% of hepatocellular carcinoma (HCC) and 4.5% of intrahepatic cholangiocarcinoma (ICC) [1, 9, 10], and the sarcomatous combined hepatocellular and cholangiocarcinoma (S-HCC–CC) is rarely published in the literature, with no more than 20 cases [11–14]. It was reported in a previous study that the PHSC carried higher aggressiveness and poorer prognosis [1, 5, 9–11, 15]. However, its prognostic significance remained unclear. Since the literature was restricted to either case reports or small case series [7, 16–20], the cross-sectional imaging features of PHSC were largely ill-defined and clinical diagnosis was difficult. The purpose of our study is to further characterize these tumors by reporting the imaging findings and clinical features of a series of 10 patients, improving the understanding and diagnosis of tumors.

## Methods

### Patient selection

We retrospectively reviewed the medical records of 13 patients with pathologically proven PHSC by fine-needle aspiration biopsy or liver resection in accordance with the World Health Organization (WHO) definition, in 2000, at our institution from January 2011 to April 2018. One patient was excluded for transcatheter arterial chemoembolization (TACE) intervention prior surgery. Extrahepatic origin of SC with liver metastasis in two patients were also excluded. The remaining 10 patients did not receive any treatment prior to the CT or MR examination. The clinical data (including demographic features, laboratory findings, clinical interventions and treatment outcomes), imaging data and pathology reports were reviewed. The study protocol was reviewed and approved by the Institutional Review Board of our hospital.

### Image acquisition

#### CT protocol

Six patients underwent Computed tomographic (CT) scans. CT scans were obtained with 64-detector row scanner (LightSpeed VCT 64, GE Healthcare, Waukesha, Wisconsin, USA). The imaging study was performed from the diaphragm to the iliac crest. The scanning parameters were as follows: tube voltage, 120 kV; tube current, 189–200 mA; matrix, 512 mm; and section thickness 5 mm. Using lopromide (Ultravist 370, Bayer Schering Pharma, Berlin, Germany) as CT contrast agent, dose: 1.5 mL/kg, injection flow rate: 3 mL/s. Finally, a 20 mL saline flush was injected at a rate of 3 mL/s. Serial dynamic contrast-enhanced scans were obtained on hepatic arterial phase (HAP) (25–40 s), portal venous phase (PVP) (45–90 s) and equilibrium phase (EP) (2–5 min) after the contrast injection by means of a bolus-triggered technique.

#### MRI protocol

Five patients underwent magnetic resonance (MR) scans. Upper abdomen MRI studies were performed on 3.0 T whole-body MRI systems (Trio, Siemens Healthineers, Erlangen, Germany) with a torso coil. The MRI protocol consisted of the following sequences and parameters: Breath-hold T1-weighted fast low angle shot sequence: TR, 170 ms; TE, 2.30 [in-phase]/3.67 ms [out-of-phase]; matrix size, 256 × 205; flip angle, 65°; T2-weighted turbo spin echo (TSE) BLADE sequence: TR, 2200 ms; TE, 103 ms; flip angle, 140°; matrix size, 320 × 106; a three-dimensional volumetric interpolated breath-hold examination (3D-VIBE) sequence was obtained before (pre-contrast) and serial dynamic contrast-enhanced scans were obtained on HAP (25–40 s), PVP (45–90 s) and EP (2–5 min) after intravenous administration of contrast agent (Gd-BOPTA, MultiHance, Bracco Pharma, Italy) at a rate of 2 mL/s for a dose of 0.1 mmol/kg body weight using a power injector. Diffusion-weighted single-shot echo-planar imaging (DWI) with simultaneous respiratory triggering was performed using TR/TE 1600 ms/76 ms. Scanning parameters were as follows: b value 0, 150, and 800 s/mm; matrix size, 192 × 144; field of view, 35 × 35 cm; number of excitations, 2; slice thickness, 5 mm; slice gap, 1 mm; and 26 axial slices.

### Image analysis

The imaging findings were evaluated as follows: 1) The location and size; 2) Gross type (nodular, massive, multinodular confluent, and infiltrative) [15]; 3) Contour (round, lobulated or irregular) and margin (sharp and indistinct); 4) Capsule (absent, complete or partial);5) Attenuation or intensity (hypo-, iso-, or hyper- attenuation or intensity relative to the background liver); 6) Lesion texture (homogeneous or heterogeneous); 7) Lesion enhancement characteristics were studied in detail as follows: the distribution in the arterial phase (peripheral rim enhancement, homogeneous enhancement and heterogeneous such as internal only or a mix rim and internal heterogeneous enhancement [21, 22]), enhancement degree of the whole tumor (heterogeneous lesions were evaluated according to the predominant parts more than 50%) [21] and the solid part only (hyperenhancement: increased enhancement compared to the background liver; hypoenhancement: decreased enhancement compared to the background liver), dynamic pattern of enhancement (partial or complete washout, progressive or persistent enhancement, no or minimal enhancement), progressive enhancement was defined as persistent or gradually increased enhancement of a tumor on portal, hepatic venous or equilibrium phase images compared with arterial phase images, as described in a previous report [23]; 8) The presence of capsule was considered positive when portal phase and equilibrium phase images demonstrated a peripheral rim of smooth hyperenhancement around the tumor [14]; and 9) Vascular invasion, intrahepatic metastasis or satellite nodule, adjacent organs invasion or seeding and lymph node metastasis. Based on these findings, image interpretation was performed retrospectively and blindly by two experienced reviewers (12 and 7 years of experience, respectively) in consensus.

### Pathology analysis

The reference standard of pathology for PHSC was based on the histopathological examination of surgical specimens for seven patients and percutaneous biopsies for three patients. The tumors were diagnosed based on the histopathologic findings and immunohistochemical results. The histopathological factors that were assessed for each tumor were as follows: gross type; histological type; fibrous capsule; necrosis or hemorrhage; vascular invasion; bile duct invasion; presence of satellite nodule or intrahepatic metastasis; and presence of extrahepatic seeding or lymph node metastasis. Microscopically, the tumors were mostly composed of pleomorphic spindle cells (sarcomatous component), and moderately to poorly differentiated adenocarcinoma or HCC component. The diagnoses and analyses were made by two senior pathologists who were in consensus.

## Result

### Baseline clinical and histological characteristics

The clinical findings are summarized in Table 1. The majority of patients in our series were male adults (nine cases), with a mean age of 53.8 years (range: 37–62 years old). The hepatitis B virus surface antigen (HBsAg) was found positive in 70% (seven of 10) of the patients and 85.7% (six of seven) of the S-HCC patients. The most common complaints were abdominal discomfort (five of 10) and fever (two of 10). Half of the patients with PHSC were detected incidentally in their routine physical examinations for the hepatitis B. The elevation of alpha-fetoprotein (AFP) was found in 50% (five of 10) of the patients and 57.1% (four of seven) of the S-HCC patients. The carbohydrate antigen 19–9 (CA19–9) was positive in 40% (four of 10) of the patients. Seven patients underwent surgical resection, and five of them were treated in combination with TACE (four cases) and radiofrequency ablation (RFA) (one case). The remaining three patients were treated with TACE (three cases) with one of them having additional percutaneous microwave ablation (PMA). According to the American Joint Committee on Cancer (AJCC) staging system of liver tumors, three patients (30%) had TNM stage I tumor, one patient (10%) had stage II, four patients (40%) had stage III, and two patients (20%) had stage IV. During the follow-up period of two-36 months, three patients with stage I had no tumor recurrence or progression, six patients with stage II-IV underwent progression, four of whom died between 2 and 5 months after diagnosis, and the remaining case with stage III did not experience any progression 1 year after the operation.

**Table 1:** Clinical findings of 10 patients with PHSC

NO.	Age (y)	Sex	Histolgic diagnosis	Confirm	Underlying disease	AFP	CA19–9	TNM^a^	Treatment after diagnosis	Follow up result
1	55	M	S-ICC	Resection	CH-A	–	–	T4N0M1	Operation	Progress (1 M) death (2 M)
2	47	M	S-ICC	Resection	CH-A	–	+	T3N0M0	Operation	Progress (2 M) death (6 M)
3	39	M	S-HCC	Resection	CH-B	+	+	T1N0M0	TACE and Operation	Tumor-free (16 M)
4	55	M	S-HCC	Biopsy	Normal	–	–	T1N0M0	TACE and RFA	Tumor-free (21 M)
5	51	M	S-HCC	Resection	CH-B	+	+	T3N1M0	Operation and TACE	Tumor-free (12 M)
6	56	M	S-HCC	Biopsy	CH-B	+	–	T3N1M1	TACE	Death (4 M)
7	56	M	S-HCC	Biopsy	CH-B	–	–	T4N1M1	TACE	Death (5 M)
8	62	F	S-HCC	Resection	CH-B	–	+	T2N0M0	Operation	Recurrence (8 M) alive (10 M)
9	60	M	S-HCC	Resection	CH-B	+	–	T1N0M0	Operation	Tumor-free (24 M)
10	57	M	S-HCC–CC	Resection	CH-B	+	–	T3N1M0	Operation and PMA	Recurrence (6 M) alive (36 M)

*+* yes/present/positive, *−* no/absent/negative

*CH-A* chronic alcoholic hepatitis, *CH-B* chronic viral hepatitis B, *CH-C* chronic viral hepatitis C, *AFP* alpha-fetoprotein (positive defined as > 7 ng/mL), *CA19–9* carbohydrate antigen 19–9 (positive defined as > 27 U/mL), *RFA* radiofrequency ablation, *TACE* transcatheter arterial chemoembolization, *PMA* percutaneous microwave ablation

^a^ AJCC TNM Staging of Liver Tumors (7th edition, 2010)

**Fig. 1:**
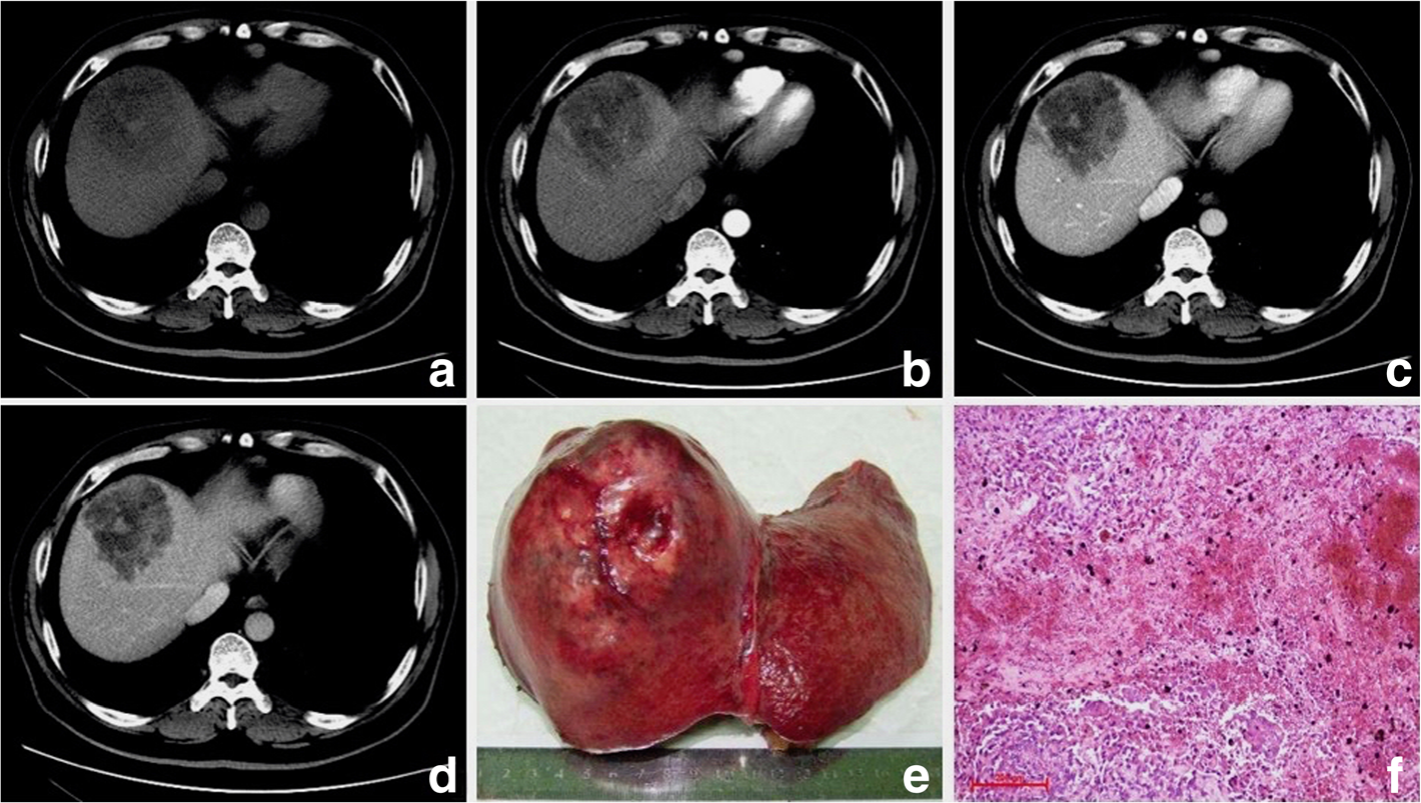
Sarcomatous intrahepatic cholangiocarcinoma in a 47 year-old man. The axial CT scans display a hypovasular mass with intratumoral hemorrhage (**a**-**d**). The bisected specimen displays a large solid tan mass with hemorrhage and necrosis breaching the liver capsule without complete penetration (**e**). Hematoxylin and eosin (H & E) stain shows multifocal hemorrhage and necrosis. Hemosiderosis is found (**f**)

**Fig. 2:**
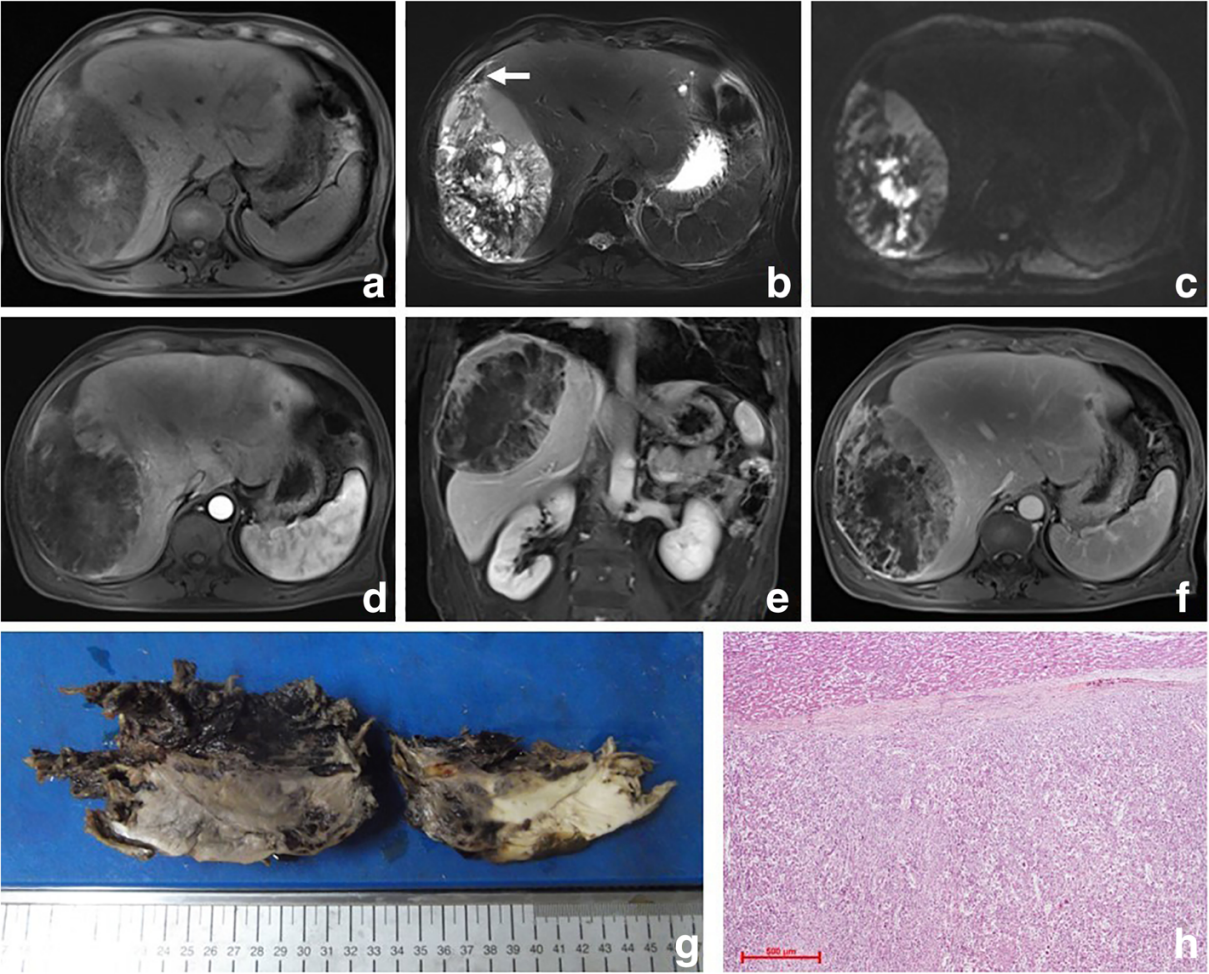
Sarcomatous intrahepatic cholangiocarcinoma in a 55 year-old man. Pre-contrast T1WI and T2WI weighted images exhibit a subcapsular mass with multilocular cystic changes and hemorrhage (**a**, **b**). The mass is inhomogeneous restricted with an obviously decreased diffusivity in the center ©. The hypo-vascular mass mainly shows irregular peripheral rim enhancement on the arterial phase (**d**), followed by centrally progressive enhancement with septa on the equilibrium phase (**e**), and capsule formation is found (**f**). The tumor is ruptured with hemorrhage found in the subcapsular space (white arrow) (**b**) and invades the diaphragm (**d**). The bisected specimen shows a fragile solid and partially necrotic white to tan mass with massive hemorrhage inside and around the tumor (**f**). H & E stain shows a fibrous capsule, separating the normal liver from tumor cells. The neoplastic cells with pleomorphism have a poor cell adhesion (**g**)

Table 2 shows pathologic features of the study population. Of the seven surgically resected tumors, the hemorrhage (three cases) and different degrees of necrosis (six cases) were found (Fig. 1). Complete and partial capsules were identified in S-ICC (Fig. 2) and S-HCC-CC respectively. Five cases involved the liver capsules (Fig. 1) with two of them (S-ICC and S-HCC) rupturing into the perihepatic space. Additional findings were vascular thrombosis (four cases), adjacent organ invasion or seeding (two cases), satellite nodules or intrahepatic metastasis (two cases), and lymph node metastasis (two cases).

**Table 2:** The histopathological findings of 7 patients with PHSC

NO.	Histological type	Fibrous capsule	Necrosis	Hemorrhage	Capsule invasion	Vascular invasion	Intrahepatic metastasis
1	S-ICC	+	+	+	+	–	–
2	S-ICC	–	+	+	+	+	+
3	S-HCC	–	N/A	N/A	–	–	–
4	S-HCC	–	+	–	+	+	+
5	S-HCC	–	+	–	+	+	–
6	S-HCC	–	+	+	+	–	–
7	S-HCC–CC	+	+	–	–	+	–

*S-ICC* sarcomatous intrahepatic cholangiocarcinoma, *S-HCC* sarcomatous hepatocellular carcinoma, *S-HCC–CC* sarcomatous combined hepatocellular and cholangiocarcinoma, *N/A* not available

### CT and MRI findings

**Fig. 3:**
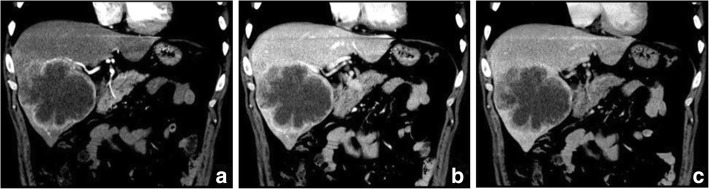
Sarcomatous hepatocellular carcinoma in a 56 year-old man. The heterogeneous mass with a large area of cystic change exhibits rim enhancement on the arterial phase (**a**) with a slight progression towards the center on the later phases (**b**, **c**)

**Fig. 4:**
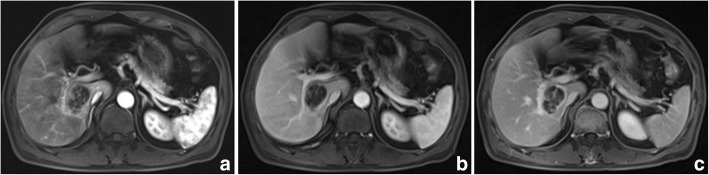
Sarcomatous hepatocellular carcinoma in a 55 year-old man. The mass shows a mix rim and internal enhancement in the arterial phase (**a**) and progressive or persistent enhancement on the portal venous and equilibrium phase (**b**, **c**). An incomplete capsule is found (**c**)

**Fig. 5:**
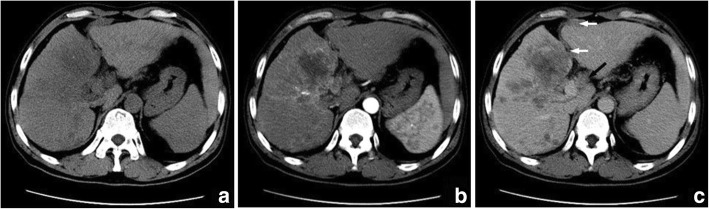
Sarcomatous hepatocellular carcinoma in a 56 year-old man. An ill-defined infiltrative mass with punctate calcification is located in the left lobe of the liver (**a**). Intrahepatic metastasis, peritoneal seeding (white arrow) and lymph node metastasis (black arrow) are found (**b**, **c**)

**Fig. 6:**
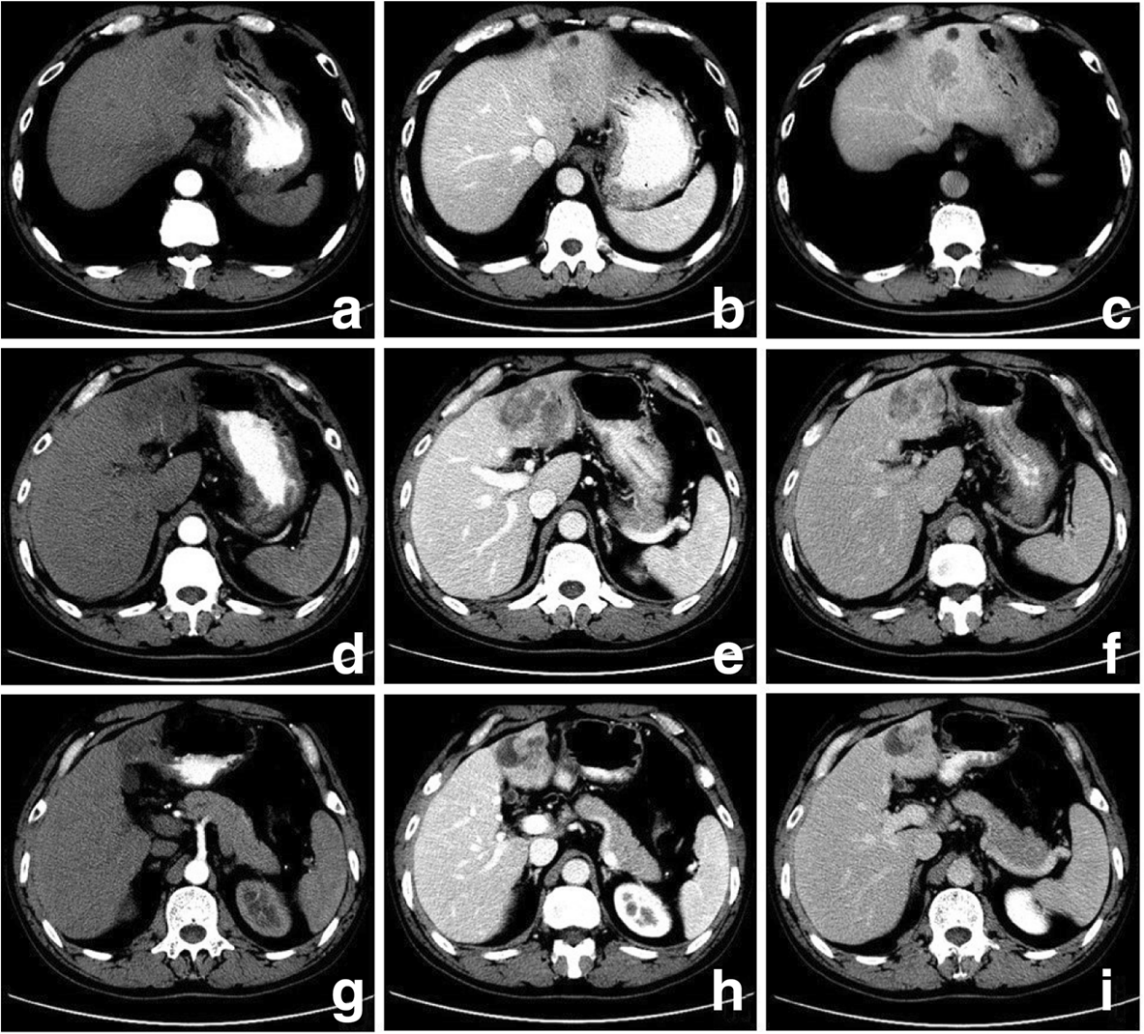
Sarcomatous combined hepatocellular and cholangiocarcinoma in a 57 year-old man. A multinodular confluent mass shows mild inhomogeneous enhancement on the arterial phase (**a**, **d**, **g**). The upper nodular shows iso-hypo enhancement on the portal venous phase (**b**) and wash out on the equilibrium phase (**c**). The lower nodular shows persistent ring enhancement with mural nodular (**h**, **i**) and the middle area shows progressive enhancement (**e**, **f**)

**Fig. 7:**
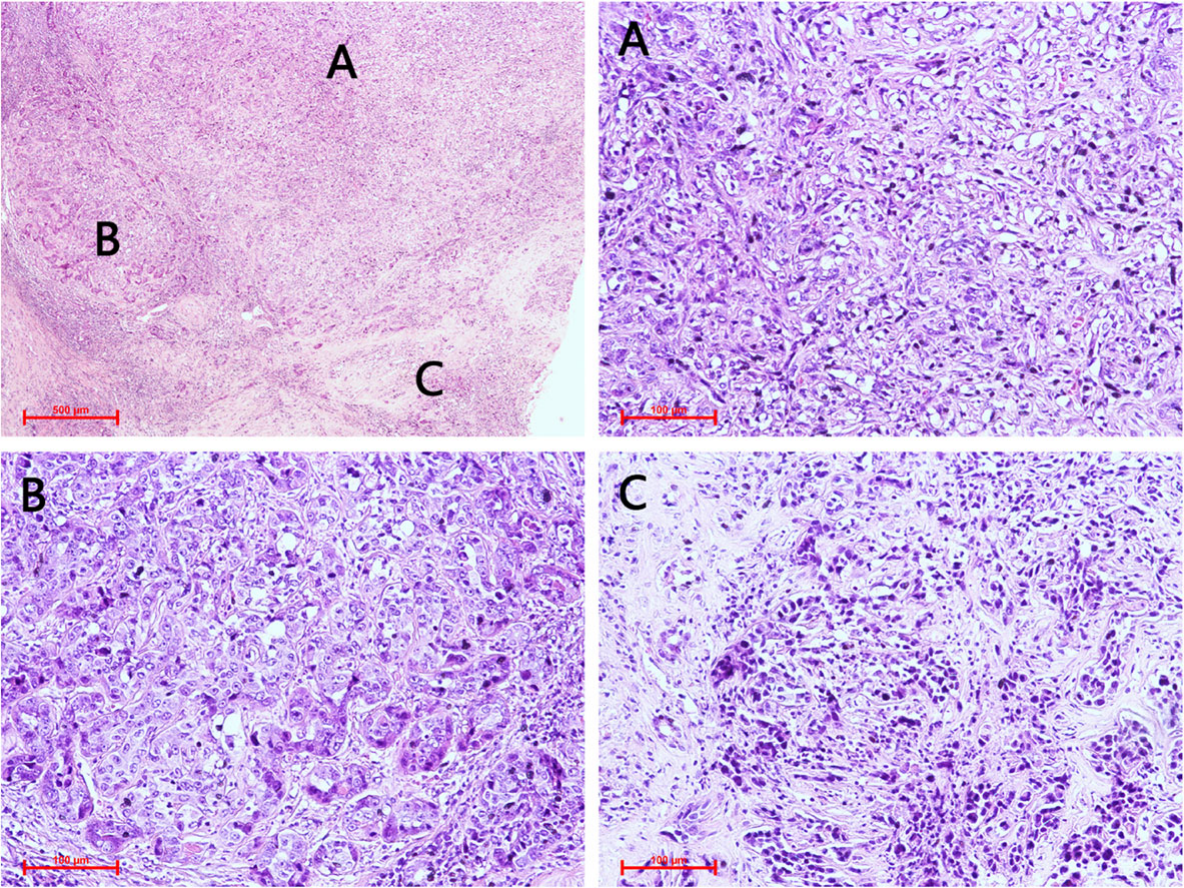
H & E stain showing tumor was composed of poor differentiated or undifferentiated sarcomatoid combined HCC and CCC (× 40). The area of sarcomatous transformation in part **a** is composed of spindle and epithelioid cells that were intensively distributed, and clumped chromatin and nucleolus can be seen (× 200); some neoplastic cells in part **b**, which are arranged in gland trabecular, strands with distinct nuclei and prominent nucleoli, are keeping with cholangiocarcinoma (× 200); some carcinoma cells with hyperchromatic nuclei and no prominent nucleoli in part **c** were arranged in sheets and strands, indicating HCC (× 200)

The imaging features of PHSC are summarized in Table 3. All of the tumors were located near the liver subcapsular area (Figs. 1, 2, 3, 4, 5, 6 and 7), with an obvious dominance (90%) in the right lobe. Tumors ranged in diameter from 3.4 cm to 22.0 cm with a mean diameter of 8.3 cm. The most common gross morphology was massive (six cases) with round or oval shapes, and the remaining included nodular (two cases), infiltrative (one case) (Fig. 5), and multinodular confluent patterns (one case) (Fig. 6). Six tumors were poorly defined, especially at CT (five cases) and the rest were demarcated at MRI. The density or signal of the tumors was mainly heterogeneous (eight cases) for hemorrhage or necrosis. The hemorrhage was found in 1S-HCC and 2 S-ICCs, with one S-ICC rupturing into the subcapsular space as shown on MR imaging (Fig. 2). Five complete or partial capsules (Figs. 2 and 4) were found on CT (one case) or MRI (four cases) and two of them were pathologically confirmed in the S-ICC and S-HCC–CC.

**Table 3:** The imaging findings of 10 patients with PHSC

NO.						Enhancement characteristics		
	Histological type	Imaging protocol	Radiological gross type	Capsule	Lesion texture	Distribution	Degree	Dynamic pattern
1	S-ICC	MR	Massive	+	Hetero	Peripheral rim	Hypo	Progressive enhancement
2	S-ICC	CT	Massive	–	Hetero	Peripheral rim	Hypo	Progressive enhancement
3	S-HCC	MR/ CT	Nodular	–	Hetero	Internal heterogeneous	Hyper	Wash out
4	S-HCC	MR	Nodular	+	Hetero	Rim and internal	Hypo	Progressive enhancement
5	S-HCC	MR	Massive	+	Homo	Internal heterogeneous	Hyper	Wash out
6	S-HCC	CT	Massive	–	Hetero	Peripheral rim	Hypo	Progressive enhancement
7	S-HCC	CT	Infiltrative	–	Homo	Peripheral rim	Hypo	Progressive enhancement
8	S-HCC	CT	Massive	–	Hetero	Peripheral rim	Hypo	Wash out
9	S-HCC	MR	Massive	+	Hetero	Rim and internal	Hypo	Progressive enhancement
10	S-HCC–CC	CT	Multinodular confluent	+	Hetero	Internal heterogeneous	Hypo	Unclassified

*S-ICC* sarcomatous intrahepatic cholangiocarcinoma, *S-HCC* sarcomatous hepatocellular carcinoma, *S-HCC–CC* sarcomatous combined hepatocellular and cholangiocarcinoma.Hetero heterogeneous, Homo homogeneous, Hypo hypoenhancement, Hyper hyperenhancement

All of the five tumors with MR examinations were mainly hypointense on T1WI and hyperintense relative to liver parenchyma on T2WI and DWI. Bright signal intensity similar to that of cyst or hemangioma on T2WI was seen in 1 S-ICC and 2 S-HCCs. The S-ICC presented multiple cystic changes accompanied by fibrous septum and was inhomogeneous restricted with an obviously decreased diffusivity in the center (Fig. 2). The signal with hyperintensity on T1WI and hypointensity on T2WI indicating hemorrhage was found not only inside the center of the tumor but also the subcapsular space (Fig. 2). The 2 S-HCCs showed homogeneous high signal on DWI and T2WI.

Regarding the distribution of enhancement, peripheral enhancement was seen in 2 S-ICCs and 3 S-HCCs (Fig. 3). Mix rim and internal heterogeneous enhancement (Fig. 4) was found in 2 S-HCCs, internal heterogeneous enhancement was shown in 2S-HCCs and 1 S-HCC–CC (Fig. 6), and no homogeneous pattern was observed. Eight tumors including 2 S-ICCs, 5 S-HCCs and 1 S-HCC–CC were hypo-vascular (Figs. 1, 2, 3 and 4) and the remaining 2 S-HCCs mainly presented hyper-intense enhancement on the arterial phase. As to the enhancement degree of the solid part in the tumor, six tumors showed hyper enhancement compared to the background liver.

With respect to the dynamic pattern of enhancement, of the ten tumors, 40% with 2 S-HCCs and 2 S-ICCs showed peripheral enhancement on the arterial phase and progressive enhancement towards the center (Fig. 2) on the later phases, 20% with 2 S-HCCs showed heterogeneous enhancement on the arterial phase and progressive or persistent enhancement on the later phase (Figs. 2, 3, 4 and 5), and 30% with 3 S-HCCs presented obvious peripheral and heterogeneous enhancement on the arterial phase and then wash out peripherally and partially on the later phase, mimicking ordinary HCC. The remaining S-HCC–CC exhibited a variable enhancement character for its multinodular change (Fig. 6). The tumor showed mild inhomogeneous enhancement on the arterial phase, and the portion near the subcapsular area of the tumor showed persistent thin rim enhancement accompanied by an mural nodular on the portal and equilibrium phase, next to the cyst change was the mild to moderate progressive fill-in enhancement, and the upper portion presented washout on the equilibrium phase.

Taking the pathology and imaging results together, five (50%) tumors including 3 S-HCCs, 1 S-ICC and 1 S-HCC–CC were accompanied by vascular invasion or thrombosis. Intrahepatic metastasis or satellite nodule was founded in 40% (4/10) tumors with 3 S-HCCs and 1 S-ICC. Extrahepatic involvement including adjacent organs invasion or seeding was observed in 1 S-ICC (Fig. 2) and 2 S-HCCs (Fig. 5), and the subcapsular rupture occurred in the S-ICC. Lymph node metastasis was found in 3 S-HCCs and 1 S-HCC–CC.

## Discussion

In agreement with previous reports, most patients (70%) in this study were found to be positive for HBsAg and negative for hepatitis C virus antigen (HCVAg), suggesting that hepatitis B virus infection might be related to PHSC [1, 5], especially to the S-HCC. Unlike S-HCCs, S-ICCs were reported to show a relatively high incidence in patients with HCV-related hepatitis as the “normal” ICCs [24, 25]. Nevertheless, the two patients with S-ICCs in our study had history of alcoholic cirrhosis without any viral hepatitis or cirrhosis. The elevation of AFP was found in 57.1% of the patients with S-HCCs, which was slightly lower than that in “normal” HCC ones. As reported in some previous studies, S-HCC was characterized by lower serum AFP level [9, 15]. Meanwhile, the opposite conclusion came from the other studies [1, 5]. Therefore, further research about the relationship among AFP, hepatitis or cirrhosis and PHSC needs to be done with a larger sample in the future.

Our study demonstrated that PHSC generally presented hypovascularity seen as peripheral enhancement on the arterial phase imaging. The PHSC was characterized by the peripheral viable cancerous tissue with fibrous stroma and central necrosis or hemorrhage [14, 26]. Similar to prior results [6, 15], the necrosis was more frequently seen with a high frequency of 85.7% (six of seven) in the surgically resected tumors in our study. The poorly differentiated cells of the sarcomatoid component grew so rapidly that the neovasculature could not adequately supply the fast-growing malignant cells, resulting in the central necrosis. In additional to the peripheral ring enhancement, when the necrosis was accompanied by fibrous septum or was scattered**,** the tumors might exhibit heterogeneous enhancement distribution such as a mix of rim and internal or internal only heterogeneous enhancement as shown in our study.

In the present study, the most common dynamic pattern of enhancement was progressive enhancement with persistent enhancement included in S-ICCs and S-HCCs. Additionally, the washout could also be found in S-HCCs. It was concluded in a previous radiologic–pathologic correlation study of S-HCC-CC that areas of arterial phase enhancement and later phase wash out were suggestive of HCC, progressive enhancements suggestive of CC, persistent and slight hypoenhancement in the subcapsular region suspicious for sarcomatoid transformation and hypo-density with little or no enhancement in keeping with the tumor necrosis [12]. Histologically, pleomorphic spindle shaped cells having loose mutual contact and fibrous stroma showed persistent or progressive enhancement, viable cells displayed a trabecular pattern with little or without fibrous stroma in the periphery of the tumor exhibited typical HCC enhancement washout, and a definite glandular pattern with fibrous stroma also showed progressive enhancement [11, 26]. Similar to the result, the S-HCC-CC in our study also presented as a lobulated multinodular confluent tumor with different dynamic enhancement patterns (Figs. 6 and 7). It was supposed that the diverse tissue composition might determine the various enhancement patterns. However, even the S-HCC exhibited different dynamic enhancement characters. So we inferred that the diverse imaging findings of the sarcomatous carcinoma not only depended on the tissue composition but also the proportion. The manifestations varied when certain histopathological components ranged from focal to prominent. Therefore, it may be necessary to further sub-classify PHSC not only using morphological criteria that define biologically distinct subgroups but also the amount of certain component, which may be related not only to the imaging findings but sometimes even the biologic behavior of these cancers [11].

On the MR imaging of five patients, bright signal intensity similar to that of cyst or hemangioma on T2WI might be explained by necrosis [14] and the signal might be attributed to hemorrhage seen as hypointensity or hyperintensity on T1WI and hypointensity on T2WI not only inside the center of the tumor but also in the subcapsular area. The other S-HCCs showed inhomogeneous high signals on DWI and T2WI, similar to the “normal” type. Five tumor capsules were observed on imaging, and only two of them were confirmed in pathology. It was reported that a high incidence was correlated with well differentiated HCC and the tumor capsules were much more common in ordinary HCC when compared with the S-HCC [5]. Similar to the result, we did not find capsules in S-HCCs, except for a complete capsule in the S-ICC and a partial one in the S-HCC-CC pathologically.

All of the PHSCs in our study located near the liver subcapsular area where the liver capsules were frequently involved (five of seven) and sometimes subcapsular metastasis or peritoneal seeding (three of 10) occurred. The invasion of the liver capsular in sarcomatous carcinoma was more common than that in the “normal” type, which might be explained by the sarcomatoid component [20]. It was found that one of mass-forming S-ICCs with a subcapsular rupture in our study protruded out of the liver contours, involve diaphragm and resulted in multifocal tumor seeding. To the best of our knowledge, four of the 30 S-ICCs reported in the English literature presented with spontaneous rupture thus far [14, 27]. The spontaneous rupture of hepatic tumor was the result of a complex interaction of various factors such as location, composition or pressure and so on [28–30]. The “normal” cholangiocarcinoma seldom ruptured spontaneously as a hard tumor with abundant fibrous stroma. Nevertheless, the S-ICC in our study presented as multiple cysts indicating more necrosis and less fibrous stroma, resulting in a fragile tumor. In comparison to the “normal” cholangiocarcinoma, the S-ICC was prone to rupture.

In additional to the liver capsular involvement found in our study, we also observed that the vascular invasion or thrombosis (50%), intrahepatic metastasis or satellite nodule (40%) were relatively common, similar to prior results [9, 16]. As for the treatment of the tumor, it was reported that radical resection at an early stage may contribute to a relatively favorable prognosis. Nevertheless, its treatment protocols and effects were still controversial. Sometimes surgical removal of the tumor alone seemed to be insufficient. The sarcomatous carcinoma was thought to be associated with aggressive tumor biology, frequent metastasis, low resectability and frequent recurrence after curative resection, segmentectomy and even liver transplantation [1, 5, 31]. The TNM stage was revealed to be one of independent risk factors for overall survival [5], which had been partially proved in our study that all deaths occurred in PHSC patients with stage III-IV between 2 and 5 months and no tumor recurrence or progression happened in the patients with stage I between 16 and 24 months.

We acknowledge several limitations to our study. The major one was its retrospective nature make the complete section-by-section matching between imaging and pathologic findings technically unfeasible. Thus, to some extent our explanations for the imaging findings might be considered speculative. Due to the retrospective nature, we were unable to categorize the amount of the certain component such as sarcomatous component and evaluated its radiological findings and prognostic significance. The second limitation was relatively small sample size, which had inherent shortcomings but was unavoidable because of the rare incidence of these tumors. The last one was that our study was only descriptive for a selected case without control group and no statistical data on differential features between them were obtained.

## Conclusion

In summary, PHSC typically manifests as subcapsular mass with hypovascularity and initial rim or heterogeneous enhancement with a dynamic progressive enhancement on later phases. They generally have a history of chronic hepatitis or cirrhosis and the treatment protocols and effect were still controversial. As a highly aggressive malignancy with more frequent vascular invasion and intrahepatic metastasis, the prognosis of PHSC was extremely poor. Given the very heterogeneous appearance of these tumors, a prospective non-invasive diagnosis based on imaging findings alone is not possible at present. Further prospective studies are warranted to enlarge the sample size, categorize the amount of certain component of the tumor and further evaluate its radiological findings and prognostic significance. Furthermore, preoperative combination therapies and postoperative adjuvant therapies in recurrent cases need to be evaluated.

## Abbreviations

AFP: Alpha-fetoprotein
CA 19–9: Carbohydrate antigen 19–9
CH-A: Chronic alcoholic hepatitis
CH-B: Chronic viral hepatitis B
CH-C: Chronic viral hepatitis C
CS: Carcinosarcoma
CT: Computed tomography
EP: Equilibrium phase
HAP: Hepatic arterial phase
MRI: Magnetic resonance imaging
PHSC: Primary hepatic sarcomatous carcinoma
PMA: Percutaneous microwave ablation
PVP: Portal venous phase
RFA: Radiofrequency ablation
RFA: Radiofrequency ablation
SC: Sarcomatous carcinoma
S-HCC: Sarcomatous hepatocellular carcinoma
S-HCC–CC: Sarcomatous combined hepatocellular and cholangiocarcinoma
S-ICC: Sarcomatous intrahepatic cholangiocarcinoma
TACE: Transcatheter arterial chemoembolization
